# A sponsorship action plan for increasing diversity in STEMM

**DOI:** 10.1002/ece3.4962

**Published:** 2019-02-10

**Authors:** Wilhelmina M. Huston, Charles G. Cranfield, Shari L. Forbes, Andy Leigh

**Affiliations:** ^1^ Faculty of Sciences, School of Life Sciences University of Technology Sydney Sydney New South Wales Australia; ^2^ Département de Chimie, Biochimie et Physique Université du Québec à Trois‐Rivières Trois‐Rivières Quebec Canada

**Keywords:** diversity and inclusion, leadership, mentor, professional development, science

## Abstract

There are numerous structural and cultural barriers to the progression of women and marginalized groups to leadership in academia, especially in Science, Technology, Engineering, Mathematics and Medicine (STEMM). A range of interventions have been described to address this inequity, with varying success. Here, we suggest that sponsorship could be one effective intervention and propose an institutional action plan to implement a sponsorship program in academia. We outline why sponsorship could be an effective strategy, especially if implemented through a deliberate program by an institution. We then detail the three components of an action plan to be considered in implementation: the elements of the program, the activities that sponsorship in academia likely encompasses, and the selection of sponsors and protégés. The plan could also be enacted by academic leadership in the absence of an institutional program and could serve as a guide to individuals in academia aspiring to address diversity and inclusion in STEMM.

## 1 INTRODUCTION

The progression of women and other marginalized groups to senior leadership roles is often hampered by unconscious bias and structural barriers, particularly in the traditional fields of Science, Technology, Engineering, Mathematics and Medicine (STEMM; Science & Gender Equity Australia, 2018). Initially, this gap was thought to be related to “the pipeline,” or a lack of girls and women wishing to enter these disciplines. However, a 2009 report from Australia found that generally <10% of academics in positions above senior lecturer (the equivalent of mid‐career professor in the US system) were women, even though 20%–50% of full‐time academic positions across the disciplines were held by women at that time (Bell, 2009). Seven years later, this vertical and horizontal gender distribution had changed little, with subtle gains at the professor level of only 20% women across STEMM fields (Science & Gender Equity Australia, 2018). We note that such analyses rarely include other marginalized groups representing diversity in a deeper sense (individuals who may base their identities on cultural background, sexuality, nonbinary gender, religion, accessibility etc), but we know there are even more barriers for these groups. What has also been evident are the increased barriers for those who have an intersectionality of identity. For many women of color, or who people who identify as LGBTIQ+, the intersectionality can not only compound the problems of career progression, but also increase the likelihood of harassment and feelings of discomfort in the STEMM workplace (Metcalf, Russell, & Hill, 2018). It is clear that women and other marginalized groups in STEMM still confront a myriad of barriers and issues to recruitment, retention, and promotion to leadership positions and further interventions are needed. Increasing diversity in academia could be addressed by targeted recruitment. However, to achieve inclusion, where the diversity is genuinely enacted by full access to opportunities by all individuals, organizational interventions are needed.

There have been numerous, different interventions initiated to attempt to achieve diversity and inclusivity in the STEMM workforce. One of the most substantial of these is the Athena SWAN program that was established in the UK in 2005 (Athena SWAN Charater, 2018). A recent survey of 4,869 academics in the UK identified that the workforce perceived that many of the barriers for women and other marginalized groups are structural and unconscious in nature (ECU, 2017). Yet, there have certainly been gains and improvement over time, suggesting that these kinds of initiatives do improve the experience for under‐represented groups. Accordingly, similar initiatives have been implemented elsewhere, such as in Australia (Science and Gender Equity in Australia SAGE; Science & Gender Equity Australia, 2018), with funding to commence a similar program announced in Canada in 2018 (Gender Equity in Canada Announcement, 2018).

In addition to these sector‐wide initiatives, there are numerous examples of approaches that have been devised to help improve inclusivity and diversity in STEMM more broadly. One example is Dame Professor Athene Donald's public advocacy for this cause, particularly her call to action through the #just1action4WIS published in the Guardian in 2015 (Donald, 2017). Other notable examples include Professor Jenny Martin's guide to achieving gender equity in conferences (Martin, 2014), Professor Imogen Coe's TEDx talk on “change the numbers” (Coe, 2016), and Professor Hilary Lappin‐Scott's work on creating a series of initiatives to have more female role models in STEM (Lappin‐Scott, 2017). More recently, the antisexual harassment movement commenced by Tarana Burke, and amplified by Alyssa Mylano with the hashtag #MeToo, has identified and enabled women in STEMM to stand up against harassment in the workplace (#MeTooSTEM; Women of Colour Magazine, 2018). Such initiatives have done much to raise awareness and prioritize ways of addressing equity and diversity in STEMM. Yet progress in academia is slow, highlighting that to overcome hidden institutional barriers will require additional explicit and proactive approaches.

## 2 LESSONS FROM THE CORPORATE SECTOR

The corporate sector has recognized the benefits to be gained from explicitly and strategically addressing gender equity and diversity issues. One intervention that has been successfully implemented is *sponsorship*. Distinct from mentorship, which is defined by psychosocial support, sponsorship is proactive and instrumental in helping to advance a career (Cao & Yang, 2013). For example, whereas a mentor will often provide counseling to a mentee, a sponsor will leverage influence and seek to provide leadership opportunities that will empower their protégé (Helms, Arfken, & Bellar, 2016). Though formal definitions of mentorship and sponsorship vary (Friday, Friday, & Green, 2004), it is generally agreed that in a mentoring relationship, the onus is on the mentee to take action in advancement of their own career. By contrast, a sponsor is more active in nominating their protégé for promotion and for prestigious roles within the institution, which then advance their protégé’s career.

Sponsorship activities have been identified as valuable and deliberately implemented in a variety of corporations including Price Waterhouse Coopers (2016) and McKinsey and Company (2018), with their global sponsorship initiative for women. These programs list sponsorship activities such as creating roles or projects for protégés, advocating for the person within the organization, enabling new roles and opportunities, directly building the protégé’s network, challenging internal politics and processes, facilitating connections to senior leaders, and providing honest feedback to the protégé. This last feature is often shared with mentorship, yet is a key element of successful sponsorship in industry: The capacity of protégés to take on critical but constructive feedback from the sponsor and develop improved professional performance as a consequence. We propose that sponsorship programs and individual sponsor activities could be better implemented in the context of academia to help improve inclusivity and diversity in our sector. We suggest this as only one action in a series of activities that should be implemented by faculties/institutions and individuals.

A feature of effective sponsorship is engagement of senior leaders who can proactively influence career opportunities and/or be more aware of such opportunities in time to assist (Cao & Yang, 2013). In our experience, sponsorship is generally more beneficial if conducted once a positive mentoring or advising relationship has already been established. We note, as identified by Laura Sherbin in the Harvard Business Review, that most successful women in STEMM are already actively sponsoring junior women in their field (Serbin, 2018).

## 3 SPONSORSHIP APPROACHES FOR ACADEMIA

Here, we provide a plan for how to sponsor and implement successful sponsorship in an academic context in order to increase gender and marginalized group diversity in academic leadership. There are two ways we suggest that sponsorship could be enacted in academia, but note they are not mutually exclusive and employing them in concert may be the most effective approach.

1. An organic approach to encouraging a selection of senior leaders in initiating their own sponsorship activities (they may already be consciously or unconsciously engaged in sponsorship).
2. A programmatic approach at the institutional level, where senior academics are identified and requested to engage in a program of sponsorship, with organizational training, professional development, and support implemented into the program. This might be termed an administrator or assessment‐based process, as outlined by Cao and Yang (2013. We propose that such a program would be resourced; would involve training in professional development, unconscious bias, and leadership for both sponsors and protégés; would potentially use an arbitrated matching process to establish sponsor‐protégé relationships; and participants would be recognized and rewarded or enabled by the institution.

Implementation of a sponsorship program involves a series of three components as outlined below. Before initiating a program, an organization should consider how to best support sponsorship (e.g., through training, incentivizing, etc.). Individuals and institutions would then benefit from curating a list of sponsorship activities. Finally, an organization should consider the attributes of sponsors and protégés that would help the selection of program participants. In the sections below, we have provided suggestions and recommendations that could assist with each stage of the planning process to ensure the goals of the program are achieved for both the sponsor and the protégé.

## 4 ELEMENTS OF AN ACADEMIC INSTITUTIONAL SPONSORSHIP PROGRAM

A sponsorship program would best work if there are support and benefits to the sponsors and protégés for participating in the program. A successful program would include explicit, organizational incentives, such as raised expectations for faculty and department level engagement, with a requirement for each faculty to have a target number of sponsor–protégé relationships enacted. Such a requirement could be supported by listing gender and marginalized group‐based sponsorship within senior staff's performance indicators. Another approach would be to ensure that sponsors are appropriately recognized and suitably rewarded for effective engagement with the program as a part of performance evaluation. Specific incentives for sponsors might include salary loadings, bonus payments, and workload allocation, or indirect incentives, such as internal strategic funding for a new direction/project or increased staffing support with additional direct reports. Such incentives are likely to be beneficial to and fully engage sponsors, especially if sponsors themselves are women or from marginalized groups.

We would recommend that to accompany a sponsorship program, the institution would actively require strong representation of different genders and marginalized peoples to provide diversity within senior management teams, institutional level committees, or governance structures that will enable opportunities for the protégés. A sponsorship program would ideally commence with training in sponsorship for both potential protégés and sponsors. A clear statement from senior leadership to these selected individuals that the institution supports the program and that development of the protégé and sponsors would be valuable. As part of this commitment, we recommend that protégés are provided access to relevant professional development and/or leadership courses during the program to ensure they are able to make the most of the opportunities that are facilitated. Given that ability to receive and apply constructive feedback is key to successful sponsorship programs in industry (Hewlett, 2013), training in reflecting on and developing from feedback should be considered. Sponsorship is inherently transactional, meaning that as protégés develop, there is an expectation of return on investment for sponsors, such as mutual advocacy, providing outcomes that validate the sponsor's efforts, and mutual growth as the sponsor is shown to grow the organization's talent (Hewlett, 2013).

We strongly recommend that participants (sponsor and protégé) in the program are offered training in unconscious bias prior to entering the program. This is important because sponsorship is subject to existing biases (conscious and unconscious) that are known to contribute to the under‐representation of women and marginalized groups in STEMM leadership positions. Unconscious bias, or implicit association (Greenwald, Banaji, & Nosek, 2018), is well documented to impact on hiring processes, including in academia (Fine et al., 2014), but biases also influence distribution of tasks and opportunities. For example, Guarino and Borden, in their evaluation of a large data set on faculties in the USA, reported that women tended to be performing more of the *internal service* or “taking care” tasks (Guarino & Borden, 2017). They defined internal service (e.g., internal governance and student administration) as the “housekeeping” and less rewarded service roles. They identified *external service* (e.g., boards, national bodies) as the beneficial, recognized, and rewarded services. This reference may be a useful resource for program managers to provide, or sponsors to use, when reflecting on or evaluating their own possible biases in recommendations of tasks to protégés. At minimum, such tendencies could be openly discussed between sponsors and protégés to ensure that both parties are aware of the breadth of potential opportunities.

To be effective in promoting minorities and women in STEMM fields, any sponsorship program should also take into account that male colleagues can and will be on the receiving end of “unofficial” sponsorship as well. This can potentially negate any significant gains an institute hopes to make in providing a more equitable workplace through any “official” sponsorship program. This fact was highlighted in a recent laboratory study that suggested that when receiving similar levels of sponsorship, males still had more competitive approaches than women (Baldiga & Coffman, 2016). Regardless, if a *targeted* programmatic sponsorship of women and marginalized groups is made, then there will be improvement in senior academic representation and achievement of these groups.

In summary, we propose that an institution should use a combination of “carrot and stick” approaches when implementing a sponsorship program, with explicit support mechanisms and requirements that enable sponsors to be active in facilitating opportunities for protégés.

## 5 WHAT ACTIVITIES CONSTITUTE SPONSORSHIP IN ACADEMIA?

For implementation of this action plan, we have constructed a list of sponsorship activities that we feel are most relevant for leaders in academia. These activities could either guide an individual taking the organic approach, or be beneficial in developing a structured program for an organization. We note that not all actions or ideas will work in all institutions and we have attempted to provide a list of possible sponsorship actions broadly applicable to academia. Sponsorship activities in academia could include the following:

- Encouraging or facilitating the protégé to apply for strategic and positive development opportunities. As highlighted above: Careful consideration is needed so that sponsors are not unconsciously aligning their protégé with under‐recognized tasks (e.g., internal service compared to external service described above).
- Notwithstanding the above, we also suggest targeting a potential protégé who has shown high effectiveness in a particular role to facilitate negotiation or realignment of their duties to roles where their strengths lie and will be showcased within the Faculty/Institution/University.
- Taking opportunities to name, positively identify and recommend the protégé and their achievements when they are not present; for example, when academic leaders are talking with their peers and put their protégé’s name forward. This is an important activity, given previous reports of a backlash effect or hidden penalties some women experience when they self‐advocate (Workplace Gender Equity Agency & A.G., 2016).
- Specifically facilitating protection of the protégé from higher risk, or less supportive members of the senior executive. Examples might include strategically selecting opportunities that only involve supportive leadership team members or actively defending the protégé in some circumstances.
- Advocating within the institution for financial support for the protégé’s development, such as professional development programs or leadership training schemes.
- Leveraging organizational commitment to support all protégés to participate in additional support programs, particularly professional coaching.
- If organizing or asked to contribute to a conference or seminar program, putting forward the protégé to be an invited speaker or session Chair. Note that opportunities such as conference organization may build the protégé’s skill set, but consideration must be given to the associated workload.
- Ensuring that the protégé is invited as a member on institutional panels or delegations to other institutions when strategic alignments are being formed.
- Introducing the protégé to key international and national level leaders showcasing their strengths; this might include a separate meeting (or informal meeting) with such people when they visit institutions, or by email.
- Introducing and ask for invited seminar opportunities for the protégé when they are traveling into an area where the sponsor has networks.
- Facilitating or providing opportunities for the person to “step up” or temporarily act in senior roles, thus developing their leadership profile.
- Providing/advocating for external opportunities, even if that results in losing the protégé from the institution. We note that losing talent is always a risk for any organization; however, in industry, where this is a commonly reported outcome of sponsorship, it is viewed as a positive (Hewlett, 2013).
- Advising on access to allowances, salary loadings, and even lobbying on behalf of the protégé to have an additional salary loading or allowance if inequities relevant to peers are apparent. This approach has actually been reported to benefit sponsorship programs in industry (Hewlett, 2013).
- Deliberately setting out to use one's leadership role or profile to create opportunities for the protégé, such as
  o introducing and recommending the protégé to industry, relevant government, or public agency representatives
  o implementing new leadership roles or committees to address needs in the institution and providing a leadership role for the protégé (e.g., deputy roles, committees around expanding areas such as diversity and inclusion).
- Ensuring that the protégé is aware of and considered for professional development opportunities, such as leadership workshops, coaching, editorial roles, board membership, membership of external bodies, or leadership roles in professional associations. This action may include the need to encourage and even assist the protégé to apply for such opportunities directly in addition to bringing it to their attention.
- Where roles tend to be based on networks and recommendations (e.g., editorial boards), advocating for the protégé to be invited to the role, even if this requires the sponsor to step down from the same board.
- Encouraging the inclusion of the protégé as an investigator on large ventures that have room for multiple participants (like large grants/projects).
- For late career professors, providing opportunities for leading postdoctoral researchers or earlier career academics to take senior authorship on research publications.
- Actively seeking to create diverse and gender‐balanced teams/research groups/committees that encourage each individual to mentor and support each other (also, as listed above, we expect there may be some explicit requirements by the institution for diversity targets for formal executive teams or committees).

## 6 SELECTION OF SPONSORS AND PROTÉGÉS

In selecting sponsors and protégés, the institution should conduct a self‐evaluation to identify key areas/faculties/departments where diversity in leadership is needed and where current leaders are likely to be capable and proactive sponsors. Effective sponsorship requires a sponsor to reflect on and consider their own motivations in order to engage with the program and protégé. Therefore, it is important to consider what might be good qualities of a sponsor and of a protégé when implementing this program to ensure it achieves the goals (Table 1 lists some of these attributes). Protégé selection could also occur during tenure review or promotion review, since these processes are established at most institutions and provide detailed information on the career progress and leadership potential of individuals. A further reason why we advocate a targeted selection of possible protégés is that, based on our experience, women and marginalized people often do not respond to general calls, but will apply when specifically approached and recommended for a program or opportunity.

**Table 1:** Key attributes to inform selection of program participants

Attributes of sponsors	Attributes of protégés
Should be senior members of the organizational team who are influential and aware of opportunities in time to strategically influence outcomes beneficial to protégés	Developing leadership potential already noted, willingness to participate in the program
Are aware of organizational strategies for strong sponsorship	Showing potential or developing engagement with organizational priorities or culture
Show professional reflection on their own strengths and challenges as well as those of the protégé to ensure that they are facilitating the most aligned opportunities	Showing capacity and willingness to receive and reflect on feedback to fully engage in professional development
Are committed to the program and protégé	
Have a track record of diverse mentorship and/or have demonstrated commitment to inclusion and diversity	Have a trajectory suggesting the potential to become a sponsor themselves as their career advances
Are able to acknowledge that not all sponsorship relationships or approaches within a relationship will work	Are able to identify members of the organization they would not feel comfortable being sponsored by
Can demonstrate a willingness to ongoing commitment, or willingness to engage with future sponsorship programs	

Organic sponsorship activities (listed as 1 above) tend to inherently arise from a past mentoring or leadership relationship with the protégé (Vries & Binns, 2018). Also, many institutions already have some kind of formal or informal mentorship program, with established partnerships in place. The existence of prior organic sponsorship relationships or mentoring arrangements could be used as part of an institutional sponsor‐protégé matching process. This is beneficial, as knowing the protégé’s preferred career directions and their strengths and weaknesses will help the sponsorship to succeed (Cao & Yang, 2013). These pre‐existing relationships might be identified by putting out a short call for interest to all senior management and leadership teams to self‐nominate as sponsors, nominate others as sponsors, and suggest protégés. Therefore, we suggest that a self and faculty level nomination process would be one ideal way to identify participants in the program.

Alternatively, an arbitrated matching process could be implemented, should faculties or departments have a number of suitable sponsors and protégés. This process should consider the strengths, challenges, and career goals of the protégés. These would then be aligned with sponsors’ strengths and challenges and, importantly, the sponsors’ capacity and circles of influence that are most relevant to the goals of the prospective protégés.

A third approach is for institutions to select developing leaders from mid‐ and early‐career academics involved in committees, and/or professional development activities as possible protégés. Similarly, leaders of committees and departments/faculties within the institution who are widely acknowledged or recognized as good mentors should be approached as sponsors. Note that in identifying potential sponsor‐protégé partnerships where the individuals belong to different faculties or disciplinary areas, consideration would need to be made of the kind of sponsorship that would help the protégé. If, for example, the protégé is in need of recognition and promotion specifically in their in STEMM field of research, then a sponsor in a STEMM faculty may be most appropriate. If, however, the protégé is showing potential and the desire for development in leadership more broadly, then a suitable sponsor might be sought from a broader pool.

## 7 CONCLUSION

In summary, we recommend specific and targeted implementation of sponsorship (as distinct from mentorship) to proactively facilitate opportunities for women and people from marginalized groups in leadership in academia. This action plan offers a way for academic institutions to implement a programmatic approach to sponsorship as one of many actions to address structural and cultural barriers to achieving diversity and inclusivity in STEMM. If, in attempting to develop a sponsor program, there is insufficient diversity of protégés considered suitable, then the structural barriers to recruitment and retention that have led to this lack of a diverse pipeline must first be addressed.

While many of the supporting studies cited above have focused on women in STEMM, we acknowledge that there are additional and greater barriers to other marginalized groups in the same environments. We recommend implementation of a sponsorship program explicitly targeting inclusion and diversity beyond gender, thereby ensuring the organization has potential to achieve a broad representation in leadership. We think the time is ripe for our sector to change, and have presented here one action plan that could be implemented among many by organizations and individuals who are committed to increasing diversity and inclusion and developing the next generation of leaders.

## CONFLICT OF INTEREST

None declared.

## AUTHOR CONTRIBUTIONS

The authors all contributed equally to constructing, drafting, and revising the manuscript. The authors all contributed to considerations of sponsorship plans in academia.

## Data Availability

This manuscript does not present any new data. The authors are happy to communicate any of their process and attempts at implementing sponsorship activities with all interested parties.
